# The H_2_S-Releasing Naproxen Derivative ATB-346 and the Slow-Release H_2_S Donor GYY4137 Reduce Intestinal Inflammation and Restore Transit in Postoperative Ileus

**DOI:** 10.3389/fphar.2019.00116

**Published:** 2019-02-20

**Authors:** Jonas Van Dingenen, Leen Pieters, Anne Vral, Romain A. Lefebvre

**Affiliations:** ^1^ Department of Basic and Applied Medical Sciences, Heymans Institute of Pharmacology, Faculty of Medicine and Health Sciences, Ghent University, Ghent, Belgium; ^2^ Department of Human Structure and Repair, Faculty of Medicine and Health Sciences, Ghent University, Ghent, Belgium

**Keywords:** ATB-346, GYY4137, hydrogen sulfide, naproxen, postoperative ileus

## Abstract

**Objective:** Intestinal inflammation triggers postoperative ileus (POI), commonly seen after abdominal surgery and characterized by impaired gastrointestinal transit; when prolonged, this leads to increased morbidity. Hydrogen sulfide (H_2_S) is recognized as an important mediator of many (patho)physiological processes, including inflammation, and is now investigated for anti-inflammatory application. Therefore, the aim of this study was to investigate the effect of the H_2_S-releasing naproxen derivative ATB-346, developed to reduce gastrointestinal injury by naproxen, and the slow-release H_2_S donor GYY4137 on intestinal inflammation and delayed gastrointestinal transit in murine POI.

**Methods:** C57Bl6J mice were fasted for 6 h, anesthetized and after laparotomy, POI was induced by compressing the small intestine with two cotton applicators for 5 min (intestinal manipulation; IM). GYY4137 (50 mg/kg, intraperitoneally), ATB-346 (16 mg/kg, intragastrically) or naproxen (10 mg/kg, intragastrically) were administered 1 h before IM. At 24 h postoperatively, gastrointestinal transit was assessed *via* fluorescent imaging, and mucosa-free muscularis segments were prepared for later analysis. Inflammatory parameters and activity of inducible nitric oxide synthase (iNOS) and cyclo-oxygenase (COX)-2 were measured. Histological examination of whole tissue sections was done on hematoxylin-eosin stained slides.

**Results:** Pre-treatment with GYY4137 (geometric center; GC: 7.6 ± 0.5) and ATB-346 (GC: 8.4 ± 0.3) prevented the delayed transit induced by IM (GC: 3.6 ± 0.5 vs. 9.0 ± 0.4 in non-operated controls) while naproxen only partially did (GC: 5.9 ± 0.5; *n* = 8 for all groups). GYY4137 and ATB-346 significantly reduced the IM-induced increase in muscular myeloperoxidase (MPO) activity and protein levels of interleukin (IL)-6, IL-1β and monocyte chemotactic protein 1; the reduction by naproxen was less pronounced and only reached significance for MPO activity and IL-6 levels. All treatments significantly reduced the increase in COX-2 activity caused by IM, whereas only GYY4137 significantly reduced the increase in iNOS activity. Naproxen treatment caused significant histological damage of intestinal villi.

**Conclusion:** The study shows that naproxen partially prevents POI, probably through its inhibitory effect on COX-2 activity. Both ATB-346 and GYY4137 were more effective, the result with GYY4137 showing that H_2_S per se can prevent POI.

## Introduction

Postoperative ileus (POI) refers to the transient impairment of gastrointestinal motility, which commonly occurs after abdominal surgery. It presents clinically as abdominal distension, inability to tolerate an oral diet, absence of bowel sounds and lack of flatus and defecation. Normally it resolves within 3 days, but when prolonged, nausea and vomiting will further contribute to increased morbidity, length of hospital stay and increased healthcare costs (Boeckxstaens and De Jonge, 2009). In the United States alone, the annual socio-economic impact of prolonged POI is estimated up to 1.46 billion US$ (Goldstein et al., 2007). POI upon intra-abdominal manipulation is induced by an acute neurogenic reaction with activation of inhibitory neural pathways and more importantly by a following prolonged inflammatory reaction triggered in the muscularis externa. Activated resident macrophages in the muscular layer play a critical role as they release inflammatory cytokines such as interleukin (IL)-6 and chemokines such as monocyte chemoattractant protein-1 (MCP-1), resulting in an increased endothelial expression of adhesion molecules such as intercellular adhesion molecule-1 and recruitment of circulatory leukocytes. Nitric oxide and prostaglandins released from these inflammatory cells will directly impair the smooth muscle contractility causing the delay in gastrointestinal transit (Boeckxstaens and De Jonge, 2009; Wehner et al., 2012). Laparoscopic procedures, enhanced recovery pathways and pharmacological treatment options such as prokinetics and non-steroidal anti-inflammatory drugs (NSAIDs) are advised to prevent prolonged POI but no full protection is obtained (Person and Wexner, 2006; Augestad, 2010; Bragg et al., 2015). Therefore, there is need for new treatment options, especially those targeting the intestinal inflammation (Van Bree et al., 2012).

Hydrogen sulfide (H_2_S), primarily known as a toxic environmental gas, is endogenously produced in mammalian tissue from cysteine, largely *via* three enzymes: cystathionine γ-lyase, cystathionine β-synthetase and 3-mercaptosulfurtransferase (Rose et al., 2017). H_2_S biosynthesis has been identified in a variety of mammalian tissues including lung, liver, heart and intestine and it has become clear that H_2_S, next to other endogenous gases like nitric oxide and carbon monoxide, plays an important role in both physiological and pathophysiological processes (Chen et al., 2007; Olas, 2015). Its role in inflammation is possibly one of the more controversial areas of the H_2_S biology as there are a lot of conflicting data concerning the pro- and/or anti-inflammatory properties of exogenous H_2_S. For example, administration of sodium hydrosulfide (NaHS), an H_2_S donor, was shown to inhibit aspirin-induced leukocyte adherence in mesenteric venules and reduced the leukocyte infiltration in an air pouch model in rats (Zanardo et al., 2006). In contrast, pre-treatment of mice with NaHS was shown to significantly enhance the lipopolysaccharide (LPS)-induced leukocyte adhesion, neutrophil migration and expression of adhesion molecules like P-selectin and intercellular adhesion molecule-1 in venular endothelium (Dal-Secco et al., 2008). One reason for the lack of clarity is the reliance on NaHS as an H_2_S donor in many studies; sulfide salts like NaHS, dissolved in aqueous solutions, will release large amounts of H_2_S within seconds (Li et al., 2008). Although the precise kinetic profile of endogenous H_2_S release within individual tissues has yet to be evaluated, it is likely that the controlled enzymatic H_2_S synthesis occurs at a much slower rate and in lesser amounts. Therefore, NaHS may not mimic the biological effects of endogenously produced H_2_S and, depending on the used dosage, may even exert toxic effects (Rose et al., 2015). In contrast to sulfide salts, GYY4137 [morpholin-4-ium 4 methoxyphenyl(morpholino) phosphinodithioate] releases H_2_S slowly both *in vitro* and *in vivo* for several hours, better mimicking the time course of naturally produced H_2_S, making this H_2_S donor more suitable to investigate the effect of exogenous H_2_S (Li et al., 2008; Lee et al., 2011). The anti-inflammatory effects of GYY4137 have already been shown in a variety of animal models including myocardial (Meng et al., 2015; Karwi et al., 2016; Qiu et al., 2018) and intestinal (Jensen et al., 2018) ischemia/reperfusion injury, LPS-induced endotoxemia (Li et al., 2009; Chen et al., 2016), atherosclerosis (Liu et al., 2013; Xie et al., 2016) and cisplatin-induced nephrotoxicity (Cao et al., 2018), whereby GYY4137 was able to reduce myeloperoxidase (MPO) activity and the level of pro-inflammatory cytokines such as IL-1β, IL-6, tumor necrosis factor-α and interferon (IFN)γ.

As mentioned earlier, NSAIDs are already used to treat pain and inflammation during prolonged POI allowing to spare opioids (Person and Wexner, 2006). However, NSAIDs, which suppress synthesis of prostaglandins by inhibiting cyclooxygenase (COX), are a major cause of gastric and duodenal ulceration and have been shown to also injure more distal parts of the small intestine, where the damage is more difficult to detect and treat (Wallace et al., 2011; Takeuchi and Satoh, 2015). It has been shown that H_2_S-releasing NSAIDs like ATB-346 [2-(6-methoxy-napthalen-2-yl)-propionic acid 4-thiocarbamoyl-phenyl ester], being the H_2_S-releasing derivative of naproxen, reduce the NSAID stimulated gastric leukocyte adhesion and protect the mucosa from ulceration (Wallace, 2007; Ekundi-Valentim et al., 2013). Moreover, released H_2_S will contribute to the overall anti-inflammatory effect of ATB-346; multiple studies have demonstrated a superior anti-inflammatory effect of ATB-346 when compared to naproxen as ATB-346 was able to reduce several inflammatory parameters like leukocyte infiltration, COX-2 activity and expression of IL-1β and tumor necrosis factor-α more effectively (Wallace et al., 2010; Campolo et al., 2013; Dief et al., 2015; Magierowski et al., 2017).

In the present study, we investigate the effect of a single dose of GYY4137 and ATB-346 on the delay in transit and inflammation during POI in mice and compare their effect with that of naproxen.

## Materials and Methods

### Materials

GYY4137 (Tocris Bioscience, Bristol, UK) was dissolved in saline (6.25 mg/ml) and ATB-346 (2 mg/ml; Axon Medchem, Groningen, Netherlands) and naproxen (1.25 mg/ml; Sigma-Aldrich, Diegem, Belgium) were suspended in 0.5% (m/v) methocel (methylcellulose, Sigma-Aldrich)/H_2_O. Isoflurane (IsoFlo®, Abbott Laboratories Ltd, Maidenhead, Berkshire, UK) was used to anaesthetize the mice when inducing POI. Fluorescein-labeled dextran (70 kDa; Invitrogen, Merelbeke, Belgium) was used to measure postoperative gastrointestinal transit.

### Animals

Seven weeks old male C57BL/6J mice were purchased from Janvier, Le Genest St-Isle, France and were used between 8 and 12 weeks of age (22–25 g). Mice were housed in an animal care facility with a 12 h light/dark cycle and had free access to water and commercially available chow. Animal care and experimental procedures were approved by the Ethical Committee for Animal Experiments from the Faculty of Medicine and Health Sciences at Ghent University (ECD 16–48).

### Surgical Procedure

POI was induced by surgical manipulation of the small intestine (intestinal manipulation, IM) as previously applied by several groups (Kalff et al., 2000; De Jonge et al., 2003; Cosyns et al., 2015). Mice were anaesthetized with inhaled isoflurane (induction, 5%; maintenance, 2%) and the abdomen was opened by midline laparotomy. The small intestine was exteriorized and then compressed for 5 min along its entire length by using sterile moist cotton swabs. The bowel was repositioned in the abdominal cavity and the incision was closed by two layers of continuous sutures. The total duration of the procedure was approximately 20–25 min. After the operation, mice were sacrificed at 24 h after induction of POI. The gastrointestinal tract from stomach until colon was then isolated. After measuring intestinal transit (see Section “Measurement of Intestinal Transit”), the gastrointestinal tract was flushed with aerated (5% CO_2_ in O_2_) ice-cold Krebs solution containing 1 mM phenylmethylsulfonyl fluoride. The small intestine was divided in 6 segments of equal length. Whole tissue sections of segment 3 were fixed in formalin for further histological examination. The mucosa was removed in the other segments using a glass slide and the muscular layer was stored at −80°C until further analysis.

### Experimental Protocol

**Figure 1:**
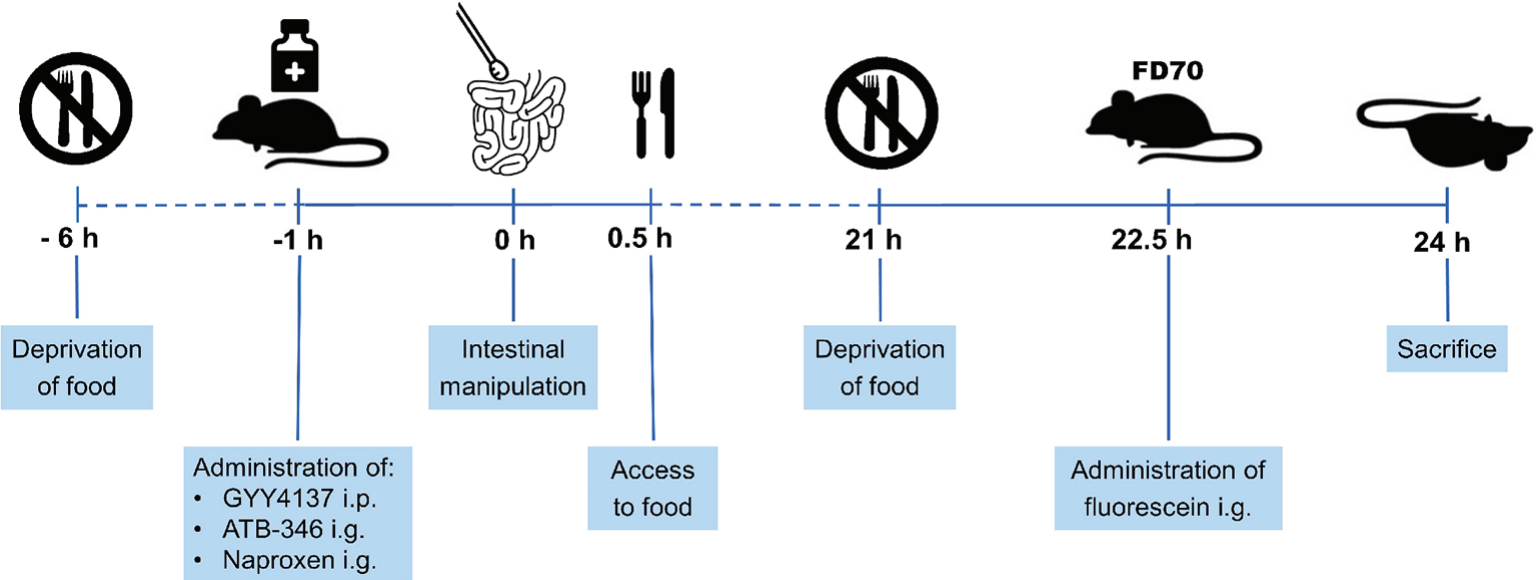
Schematic overview of experimental protocol. Mice were deprived of food from 6 h before intestinal manipulation (IM) and given GYY4137, ATB-346 or naproxen 1 h before IM. When mice recovered from surgery, they had again access to food. Mice were deprived of food at 21 h after IM and received 200 μl of fluorescein-labeled dextran (FD70) at 22.5 h after IM. 1.5 h later, mice were sacrificed followed by measurement of the gastrointestinal transit and collection of tissue samples of the small intestine.

Mice were randomly assigned to five experimental groups (*n* = 8 per group). Group I served as control (non-treated, non-operated). Group II underwent surgical manipulation of the small intestine (IM). Group III to V received respectively GYY4137 intraperitoneally (i.p., 50 mg/kg), naproxen intragastrically (i.g., 10 mg/kg) and ATB-346 i.g. (16 mg/kg) at 1 h before the surgical procedure. All animals were deprived of food from 6 h before IM but had again access to food after IM. At 21 h after surgery, mice were again deprived of food to allow measurement of intestinal transit (see Section “Measurement of Intestinal Transit”). Mice were sacrificed at 24 h after surgery. Figure 1 shows a schematic overview of the protocol.

### Measurement of Intestinal Transit

Intestinal transit was measured by evaluating the distribution of non-absorbable fluorescein-labeled dextran (70 kDa; FD70) along the gastrointestinal tract as previously described (De Backer et al., 2008). 21 h after IM, the mice were deprived of food and 1.5 h hereafter a liquid fluorescein-labeled dextran meal (200 μl of a 25 mg/ml solution) was gavaged. 1.5 h later, i.e. 24 h after surgery, the animals were sacrificed. After excision of the gastrointestinal tract, two full-field images (one in normal illumination mode and another in fluorescent mode) were taken with a CCD camera and subsequently matched for calculation of fluorescence distribution along the gastrointestinal tract. The fluorescent intensity throughout the entire gastrointestinal tract was analyzed and calculated. Data were expressed as the percentage of fluorescence intensity per segment (stom, stomach; sb, small bowel segments 1–10; caec, caecum; col, colon segments 1–2). The geometric center (GC) of the fluorescence distribution, which can be described as the center of gravity for the distribution of fluorescein, was calculated by the formula: ∑(% FD70 per segment*segment number)/100). It can range from 1, if all fluorescein were found in the stomach, to 14, if all fluorescein were found in the second segment of the colon.

### Biochemical Analyses

#### MPO Activity

MPO activity in mucosa-free segments of the small intestine was measured as an index of neutrophil infiltration, based on a previously described protocol (De Jonge et al., 2003).

Frozen tissue samples were homogenized with a Mikro-Dismembrator and dissolved in 10 volumes of 50 mM potassium phosphate buffer (pH 6.0) containing 0.5% hexadecyl-trimethylammonium bromide (HETAB). The homogenate was sonicated on ice (15 pulses of 0.7 s at full power) and subsequently subjected to freeze/thaw. The suspension was centrifuged (14,000 *g*, 20 min, 4°C) and 10 μl of the supernatant was added to 200 μl of assay mixture, containing ready-to-use 3,3′,5,5′-tetramethylbenzidine substrate, 0.5% HETAB, and 10 mM EDTA (on ice). The optical density was immediately read at 620 nm (Biotrak II). The reaction was then allowed to proceed for 3 min at 37°C. The reaction was stopped by placing the 96-well plate on ice, and the optical density was measured again. One unit of MPO activity was defined as the amount of enzyme that produces a change in optical density of 1.0 per minute at 37°C. Results were normalized to total protein content (Pierce BCA Protein Assay Kit) and expressed as U/mg protein.

#### IL-1β, IL-6, IL-10, IFNγ and MCP-1 Protein Levels

Protein expression levels of IL-1β, IL-6, IL-10, IFNγ and MCP-1 in mucosa-free segments of the small intestine were assessed by a Luminex magnetic bead assay according to the manufacturer’s guidelines (Bio-Rad, Temse, Belgium); the samples were processed using the BioPlex Pro Reagent Kit (Bio-Rad).

Frozen tissue samples were homogenized in PBS, containing protease and phosphatase inhibitors. The homogenate was sonicated on ice (5 pulses of 0.7 s) and after centrifugation (15,000 rpm, 15 min, 4°C), 50 μl of the supernatant was added to the corresponding well of a 96-well plate loaded with coupled beats for IL-1β, IL-6, IL-10, IFNγ and MCP-1. After placing the plate for 30 min on a shaking platform at 850 rpm protected from light, wells were washed and 25 μl of the mixture of detection antibodies for IL-1β, IL-6, IL-10, IFNγ and MCP-1 was added to each well. After placing the plate for another 30 min on the shaking platform at 850 rpm protected from light, wells were washed and 50 μl of Streptavidin Phycoerythrin solution was added to each well. After placing the plate for 10 min on the shaking platform at 850 rpm protected from light, wells were washed and the beads in each well were re-suspended in 125 μl assay buffer. After a last incubation step of 30 s, the wells were analyzed for IL-1β, IL-6, IL-10, IFNγ and MCP-1 protein concentration using Bio-Plex Manager Software (Bio-Rad). Data from tissue samples were normalized to the total protein content (Pierce BCA Protein Assay Kit).

#### COX-2 Activity

COX-2 enzyme activity was measured in mucosa-free segments by COX activity assay (Cayman Chemical) using arachidonic acid as a substrate and 10-acetyl-3,7-dihydroxy-phenoxazine (ADHP) as a fluorometric co-substrate. The reaction between COX-derived prostaglandin G2 and ADHP produces the highly fluorescent compound resorufin, which was measured.

Frozen tissue samples were homogenized with a Mikro-Dismembrator, dissolved in 5 volumes of 100 mM Tris-HCl buffer (pH 7.5), containing protease inhibitors, and centrifuged at 10,000 *g* for 15 min at 4°C. 10 μl of the supernatant was transferred to the appropriate well containing 140 μl of 1X Assay Buffer (100 mM Tris-HCl, pH 8.0), 10 μl of Heme in DMSO and 10 μl of the COX-1 inhibitor SC-560 (66 μM). After an incubation period of 5 min, 10 μl of ADHP Assay Reagent was added to each well. Administration of 10 μl of a 2 mM arachidonic acid solution to each well initiated the reaction; 1 min later the plate was measured using a spectrofluorometer (excitation wavelength: 530–540 nm, emission wavelength: 585–595 nm; Victor Wallac, PerkinElmer Life and Analytical Sciences). Background values were measured by not adding 10 μl of the arachidonic acid solution to the assigned wells and were deducted from the corresponding sample values. Results were normalized to total protein content (Pierce BCA Protein Assay Kit) and COX-2 activity was expressed as % of mean fluorescence in tissue from non-manipulated control mice.

#### iNOS Activity

Inducible NO synthase (iNOS) enzyme activity in intestinal muscularis samples was assayed by measuring the conversion of [^3^H]-arginine to [^3^H]-citrulline by use of a NOS activity assay (Cayman Chemical), according to the manufacturer’s protocol. The assay was conducted in calcium-free conditions to measure only iNOS.

Frozen tissue samples were homogenized with a Mikro-Dismembrator, dissolved in five volumes of 1X Homogenization Buffer and centrifuged at 10,000 *g* for 15 min at 4°C. 10 μl of the supernatant was added to 40 μl reaction mix (22.72 μl of Reaction Buffer [50 mM Tris-HCl (pH 7.4), 6 μM tetrahydrobiopterin, 2 μM flavin adenine dinucleotide, 2 μM flavin adenine mononucleotide], 4.55 μl of 10 mM NADPH (prepared in 10 mM Tris-HCl), 0.90 μl of [^3^H]-arginine (1 μCI/μl), 4.55 μl 8 mM Mg acetate, 3.64 μl calmodulin and 3.64 μl ddH_2_O). The reaction samples were incubated for 1 h at room temperature, and the reaction was stopped by adding 400 μl of Stop Buffer to the reaction sample. 100 μl of the equilibrated resin was added into each reaction sample and the reaction samples were then transferred in the provided spin cups, which were centrifuged for 30 s in a microcentrifuge at full speed. The eluate was then transferred to scintillation vials, and, after adding 2 ml scintillation solution (Ultima Gold, Canberra Packard, USA) to each vial, the radioactivity was quantified in a liquid scintillation counter (Packard Tri-Carb 2,100 TR, Canberra Packard, USA). Results were normalized to total protein content (Pierce BCA Protein Assay Kit) and iNOS activity was expressed as % of [^3^H]-citrulline in tissue from non-manipulated control mice.

### Intestinal Histological Evaluation

Intestinal samples were fixed in formalin, dehydrated in a graded ethanol series and embedded in paraffin. Tissue sections of 5 μM were cut with a microtome and then stained with hematoxylin-eosin. Jonas Van Dingenen and Leen Pieters independently scored the slides to evaluate intestinal mucosal damage using the Chiu score (Chiu et al., 1970): grade 0, normal mucosa villi; grade 1, development of sub-epithelial Gruenhagen’s space at the apex of the villi; grade 2, extension of the sub-epithelial space with moderate epithelial lifting; grade 3, massive epithelial lifting, possibly with a few denuded villi; grade 4, denuded villi with lamina propria and exposed capillaries; and grade 5, disintegration of the lamina propria, ulceration and hemorrhage. For each sample, 100 villi were scored by both evaluators and the mean score per sample was calculated. Good correlation between the scores of the two evaluators is illustrated by a Pearson’s r-value of 0.8324.

### Statistical Analysis

Data are given as mean ± s.e.m. as indicated; n refers to tissues obtained from different animals. The results were compared by one-way analysis of variance (ANOVA), followed by Bonferroni multiple comparison t-test for all manipulated groups vs. the non-operated control group and for the treated manipulated groups vs. the non-treated manipulated group. Results were considered different from a *p* less than 0.05 on (GraphPad version 5.03, San Diego, CA, USA). The Grubbs’ test was used to determine significant outliers (*p* < 0.05), that were excluded for further statistical analysis (Grubbs, 1969). The Pearson correlation test was used to investigate the correlation between the scores of the two evaluators performing the intestinal histological evaluation.

## Results

### Effect of GYY4137, Naproxen and ATB-346 on Manipulation-Induced Intestinal Dysmotility

**Figure 2:**
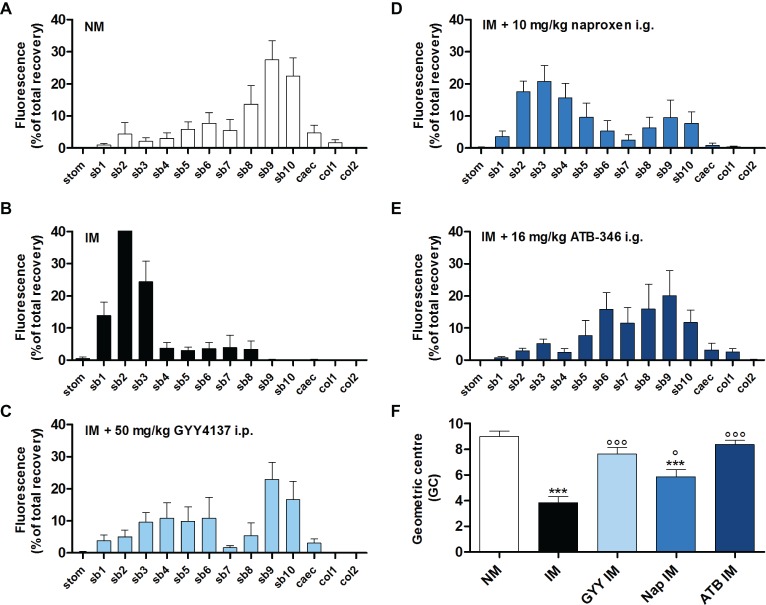
Influence of GYY4137, naproxen and ATB-346 on the delay in gastrointestinal transit caused by intestinal manipulation. Transit histograms **(A–E)** and geometric center **(F)** for the distribution of fluorescein-labeled dextran (70 kDa) along the gastrointestinal tract (stom, stomach; sb, small bowel segments; caec, caecum; col, colon segments), measured 24 h after intestinal manipulation (IM). Data represent the means ± S.E.M. of *n* = 8. ***p < 0.001 for comparison with non-manipulated (NM) control mice; °*p* < 0.05, °°°*p* < 0.001 for comparison with non-treated IM mice: one-way ANOVA followed by a Bonferroni multiple comparison test.

In non-manipulated control mice, fluorescein-labeled dextran had mainly moved to the distal part of the small bowel (Figure 2A). In mice that underwent intestinal manipulation, fluorescein-labeled dextran was mostly present in the proximal part of the small bowel, pointing out a delay in transit (Figure 2B), which was confirmed by a significant reduction in GC (Figure 2F). Pre-treatment with 50 mg/kg GYY4137 i.p. or 16 mg/kg ATB-346 i.g. reduced the delay in transit caused by IM, as indicated by the shift of fluorescein-labeled dextran to the distal part of the small bowel (Figures 2C–E); the GC was significantly increased vs. that in untreated manipulated animals and no longer significantly different from that in non-manipulated control mice (Figure 2F). Pre-treatment with naproxen did not completely prevent the delay in transit caused by IM as the GC-value was still significantly different from that in non-manipulated control mice (Figure 2F).

### Effect of GYY4137, Naproxen and ATB-346 on Manipulation-Induced Inflammation

**Figure 3:**
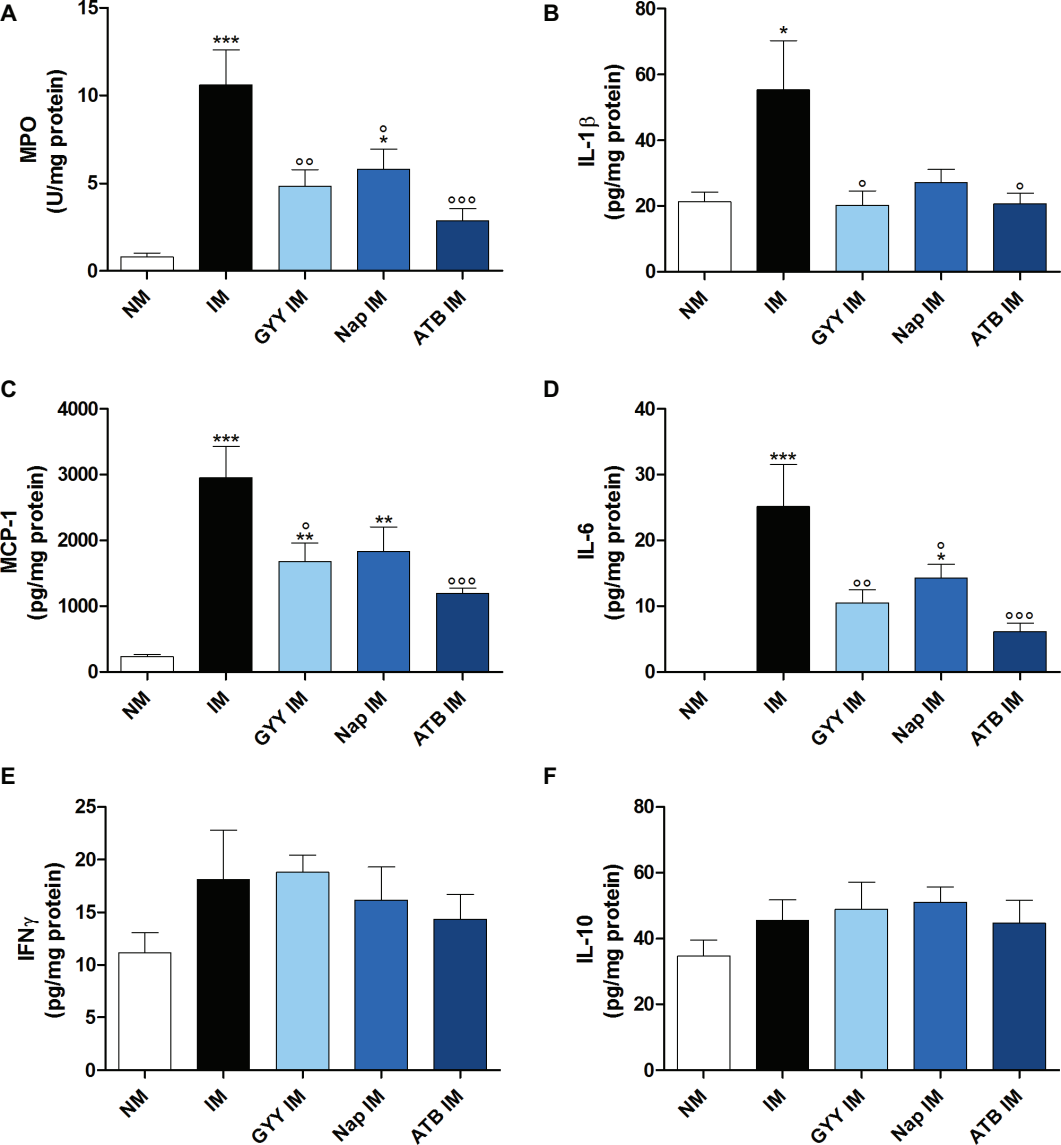
Influence of GYY4137, naproxen and ATB-346 on intestinal manipulation induced inflammation. Leukocyte infiltration (**(A)**; myeloperoxidase activity, MPO) and IL-1β **(B)**, MCP-1 **(C)**, IL-6 **(D)**, IFNγ **(E)** and IL-10 **(F)** protein levels in the muscular layer, measured 24 h after intestinal manipulation (IM). Data represent the means ± S.E.M. of *n* = 7–8. *p < 0.05, **p < 0.01, ***p < 0.001 for comparison with non-manipulated (NM) control mice; °*p* < 0.05, °°*p* < 0.01, °°°*p* < 0.001 for comparison with non-treated IM mice: one-way ANOVA followed by a Bonferroni multiple comparison test.

The leukocyte infiltration into the muscularis, measured as MPO activity, was increased 24 h after IM. This IM-induced accumulation of leukocytes in the muscular layer was significantly reduced when mice were pre-treated with 50 mg/kg GYY4137 i.p., 10 mg/kg naproxen i.g. or 16 mg/kg ATB-346 i.g. (Figure 3A). However, the MPO activity in the naproxen treated group was still significantly increased compared to the non-manipulated control group.

As shown in Figures 3B–D, surgical manipulation of the small intestine markedly increased IL-1β, MCP-1 and IL-6 protein expression in the intestinal muscular layer at 24 h after IM. Pre-treatment with all three compounds resulted in a significant reduction of the elevated IL-6 levels but the level of IL-6 in the group treated with naproxen remained significantly different from that in non-manipulated control animals (Figure 3D). Only GYY4137 and ATB-346 were able to significantly reduce the increase in IL-1β and MCP-1 levels caused by IM (Figures 3B,C). No significant increase in protein expression of IL-10 and IFNγ was observed in the muscularis of manipulated mice with the Luminex magnetic bead assay (Figures 3E,F).

### Effect of GYY4137, Naproxen and ATB-346 on Intestinal iNOS and COX-2 Activity

**Figure 4:**
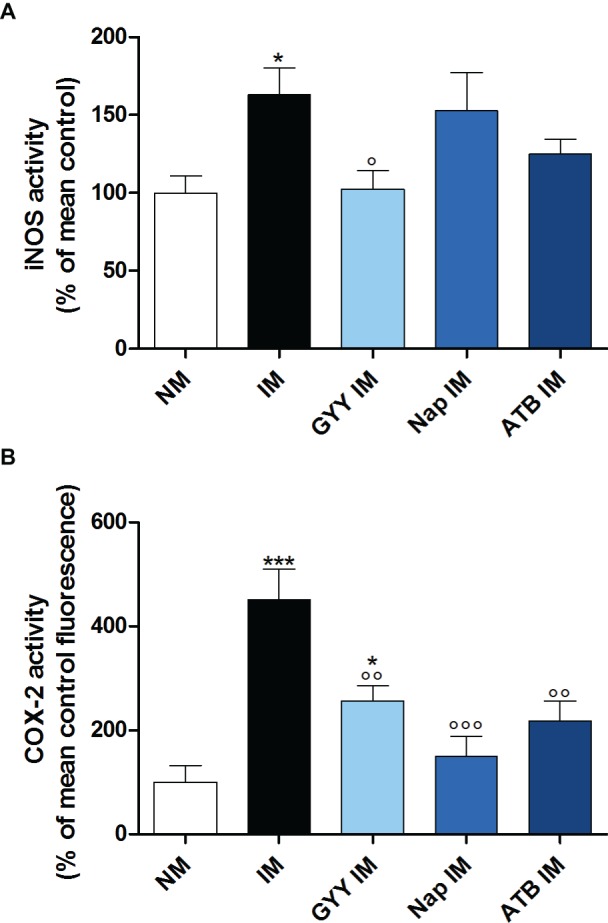
Influence of GYY4137, naproxen and ATB-346 on intestinal iNOS and COX-2 activity. iNOS **(A)** and COX-2 **(B)** activity in the muscular layer, measured 24 h after intestinal manipulation (IM). Data represent the means ± S.E.M. of *n* = 8. *p < 0.05, ***p < 0.001 for comparison with non-manipulated (NM) control mice; °*p* < 0.05, °°*p* < 0.01, °°°*p* < 0.001 for comparison with non-treated IM mice: one-way ANOVA followed by a Bonferroni multiple comparison test.

Surgical manipulation of the small intestine caused a significant increase in iNOS enzyme activity in the muscularis. Only GYY4137 significantly reduced this increase in iNOS activity. Treatment with ATB-346 also tended to reduce the elevated iNOS activity, but this effect was not significant (Figure 4A). Surgery also caused a pronounced increase in COX-2 activity. All three compounds were able to significantly reduce the increased COX-2 activity caused by IM (Figure 4B).

### Effect of GYY4137, Naproxen and ATB-346 on Morphology of Intestinal Villi

**Figure 5:**
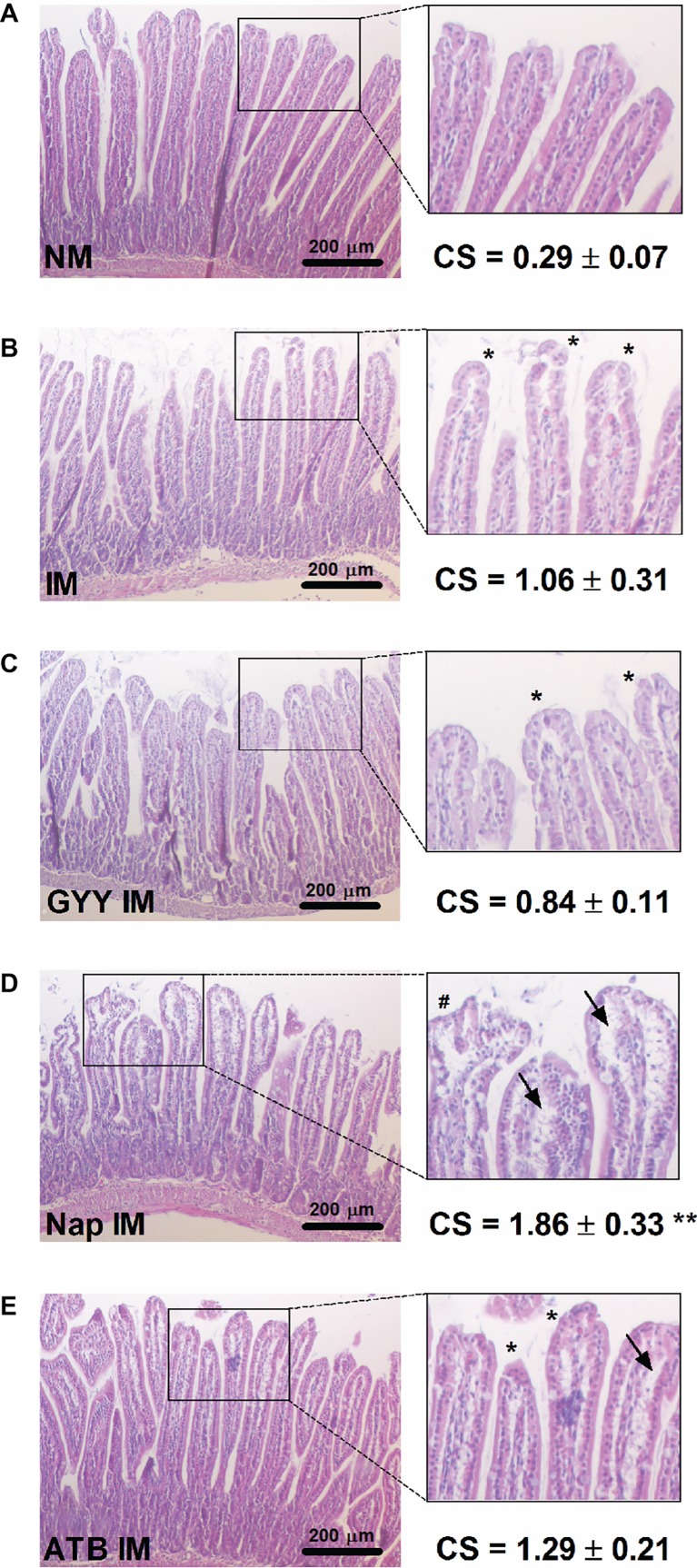
Influence of GYY4137, naproxen and ATB-346 on morphology of intestinal villi. Histological analysis and Chiu score (CS;**A–E**) of small intestinal mucosa following hematoxylin-eosin staining, analyzed 24 h after intestinal manipulation (IM). Asterisks (*) mark the development of sub-epithelial Gruenhagen’s space at villus apex; arrows (→) indicate further epithelial lifting down the sides of the villi and the number signs (#) mark massive epithelial lifting. Data represent the means ± S.E.M. of *n* = 4. **p < 0.01 for comparison with non-manipulated (NM) control mice: one-way ANOVA followed by a Bonferroni multiple comparison test.

No injury was seen in the non-manipulated control group, which demonstrated normal mucosal villi (Figure 5A). However, in the non-treated (Figure 5B) and GYY4137 treated (Figure 5C) manipulated mice, development of sub-epithelial Gruenhagen’s space in the tip of the villi was observed although the increase in Chiu score did not reach significance. The intestinal structure was damaged most severely in the naproxen treated IM group (Figure 5D), regularly showing moderate to massive epithelial lifting down the sides of the villi leading to a significant increase in Chiu score. Compared to the naproxen treated IM group, villi were less damaged when animals were treated with ATB-346 (Figure 5E), showing Gruenhagen’s space at the apex of the villi and moderate epithelial lifting.

## Discussion

Hydrogen sulfide therapy has attracted significant attention as a potent biological mediator. Although its benefits have been appreciated in different gastrointestinal disease models like colitis (Fiorucci et al., 2007; Chen and Liu, 2016) and intestinal ischemia/reperfusion injury (Henderson et al., 2010; Jensen et al., 2018), its potential effect on POI has not yet been investigated. POI is a serious complication seen after abdominal surgery, leading to impairment of the gastrointestinal transit that traditionally was thought to result from neuronal dysfunction. Presently, it is clear that the immune response in the intestinal muscular layer upon abdominal surgery, characterized by the activation of resident macrophages and upregulation of inflammatory cytokines, adhesion molecules and chemokines, is the main mechanism of POI (Boeckxstaens and de Jonge, 2009; Van Bree et al., 2012; Bragg et al., 2015). Many of the data leading to the pathophysiological explanation of POI were obtained in the murine model of POI with manipulation of the small intestine. Throughout the years, this model yielded consistent results for transit and inflammation in our and other laboratories (Moore et al., 2003; Nakao et al., 2006; De Backer et al., 2009; Engel et al., 2010; Snoek et al., 2012; Cosyns et al., 2015). Also in the present study an inflammatory response is observed after manipulation of the murine small intestine, as shown by the increased leukocyte infiltration and elevated protein levels of the pro-inflammatory cytokines IL-6 and IL-1β and the chemokine MCP-1 in the intestinal muscularis externa at 24 h after induction of POI. Although previous reports (De Backer et al., 2009; Engel et al., 2010) show elevated IL-10 protein and IFNγ mRNA levels at 24 h after IM, we could not measure a significant increase in protein expression for these cytokines with the Luminex magnetic bead assay used in this study.

GYY4137 is an H_2_S-releasing molecule that was shown to have a pronounced anti-inflammatory effect in rodent models of myocardial (Qiu et al., 2018) and intestinal (Jensen et al., 2018) ischemia/reperfusion injury, endotoxemia (Chen et al., 2016) and atherosclerosis (Liu et al., 2013). The usual dose applied is 50 mg/kg by i.p. route (Liu et al., 2013; Chen et al., 2016; Jensen et al., 2018; Qiu et al., 2018). I.g. administration of GYY4137 has to be avoided since the release of H_2_S from GYY4137 will be enhanced under acidic conditions (Li et al., 2008), possibly leading to toxic H_2_S levels as seen with fast-release H_2_S donors such as NaHS. H_2_S-releasing naproxen ATB-346 showed beneficial anti-inflammatory effects in rodent models of colorectal cancer (Elsheikh et al., 2014), melanoma (De Cicco et al., 2016), zymosan-induced arthritis (Dief et al., 2015), spinal cord injury (Campolo et al., 2013) and carrageenan-induced synovitis (Ekundi-Valentim et al., 2013); the usual dose is 16 mg/kg by i.g. route (Ekundi-Valentim et al., 2013; Elsheikh et al., 2014; Dief et al., 2015). The studied route of administration for ATB-346 in rodents is indeed the i.g. one, as the compound is developed to improve the efficacy and decrease the side effects of the NSAID naproxen, used perorally in clinical practice. As neither GYY4137 nor ATB-346 has been tested before for POI, this was done in the actual study. I.g. naproxen was studied in comparison with ATB-346; the dose used (10 mg/kg) is the molar equivalent of the amount of naproxen contained within 16 mg/kg ATB-346 and has already been shown to exert anti-inflammatory effects in a rat model of carrageenan-induced synovitis (Ekundi-Valentim et al., 2013).

In the present study we show that both GYY4137 and ATB-346 were able to completely reverse the delay in gastrointestinal transit seen after IM, whereas naproxen was only able to partially restore the delayed transit. The findings for the NSAID naproxen are similar to previous work, in which the authors show the beneficial effects of pre-treatment with the non-selective COX inhibitors indomethacin and ketorolac and the selective COX-2 inhibitor 5,5-dimethyl-3-(3-fluorophenyl)-4-(4-methylsulphonyl) phenyl-2(5H)-furan on the impaired transit in a rat model of POI (De Winter et al., 1998; Schwarz et al., 2001). Treatment with GYY4137 and ATB-346 was also found to reduce the IM-induced inflammatory response, as evidenced by a marked reduction of the inflammatory mediators IL-1β, IL-6 and MCP-1 and leukocyte infiltration; whereas the reduction caused by naproxen only reached significance for IL-6 protein levels and leukocyte infiltration. These data are consistent with previous evidence showing that the H_2_S-releasing moiety of ATB-346 adds complimentary anti-inflammatory effects to the COX inhibiting effect of naproxen (Wallace et al., 2010; Campolo et al., 2013; Dief et al., 2015; Magierowski et al., 2017). The functional importance of the kinetically active substances NO and prostaglandins, that directly inhibit smooth muscle contractility, in the pathogenesis of POI has been demonstrated in earlier studies (Kalff et al., 2000; Schwarz et al., 2001). Therefore, we further investigated whether GYY4137, naproxen or ATB-346 could alter iNOS and COX-2 enzyme activity levels. Only GYY4137 was able to significantly reduce both the increased iNOS and COX-2 activity. Similarly, in an *in vitro* study in human synoviocytes and articular chondrocytes GYY4137 was able to dose-dependently reduce the LPS-induced increase in both iNOS and COX-2 expression and activity (Li et al., 2013). ATB-346 and naproxen did only cause a significant reduction in COX-2 activity, although ATB-346 tended to reduce iNOS activity. These findings are in line with the study of Campolo et al. in which ATB-346, but not naproxen, was able to attenuate iNOS expression in a murine model of spinal cord injury; it has to be noted that naproxen did also not affect COX-2 expression in this model (Campolo et al., 2013). However, the same research group also reported that in a model of traumatic brain injury in mice, administration of both ATB-346 and naproxen led to a significant reduction of COX-2 and iNOS expression (Campolo et al., 2014). The absence of any effect with naproxen on iNOS activity in the actual study might contribute to the less pronounced effect of naproxen on retarded transit.

As mentioned earlier, NSAIDs are a major cause of gastric and duodenal ulceration and have been shown to also injure more distal parts of the small intestine. The protective effect of H_2_S-releasing NSAIDs like ATB-346 on gastric and intestinal lesions has been well described in the literature and a double-blind, controlled clinical study to compare the gastrointestinal safety of ATB-346 vs. naproxen in healthy subjects is ongoing (ClinicalTrials.gov^1^; Wallace, 2007; Wallace et al., 2010; Magierowski et al., 2017). In this study intestinal manipulation per se tended to induce moderate mucosal damage although not reaching significance; a similar Chiu score was seen in manipulated mice pretreated with GYY4137. In manipulated mice, pretreated with naproxen, a significant increase in Chiu score was seen, confirming the well-known intestinal mucosal injury by NSAIDs. However in manipulated mice pretreated with ATB-346, the Chiu score was similar to that in non-treated manipulated mice showing that the H_2_S-release from this molecule was able to counteract mucosal injury of the naproxen moiety.

Follow-up studies can be performed to unravel the underlying mechanisms by which these H_2_S-releasing compounds exert their beneficial effects in the murine model of POI. The most frequently described mechanisms by which exogenous H_2_S exerts its anti-inflammatory effect are inhibition of the pro-inflammatory nuclear transcription factor-κB (Oh et al., 2006; Li et al., 2013; Chen and Liu, 2016), activation of the anti-oxidant nuclear factor erythroid 2–related factor 2 (Shimada et al., 2015; Xie et al., 2016; Qiu et al., 2018) and inhibition of the mitogen-activated protein kinase signaling pathways like p38 and extracellular signal-regulated kinase 1/2 (Hu et al., 2007; Xu et al., 2013). Furthermore, it can be investigated whether the endogenous H_2_S pathway is upregulated during POI as a protective mechanism, whereby exogenous H_2_S donors then strengthen this reaction; enhanced H_2_S synthesis with increased expression of H_2_S synthetizing enzymes at sites of mucosal ulceration was indeed demonstrated in rat colitis (Wallace et al., 2009; Flannigan et al., 2013).

This study demonstrates that pre-treatment with GYY4137, ATB-346 or naproxen reduces the impaired transit and suppresses the IM-induced inflammatory response in the muscularis seen during POI in mice, with naproxen being the least effective compound. Furthermore, the intestinal mucosal damage with naproxen alone was prevented by administering it as a H_2_S-releasing compound. The data suggest that delivery of H_2_S may represent a new pharmacological approach to prevent POI or to improve the effect of NSAIDs in POI. NSAIDs are indeed already studied for postoperative ileus with some improvement of time to gut recovery (Milne et al., 2018). The current data suggest that ATB-346 is preferable over naproxen per se for application in POI. The step to a clinical trial with ATB-346 to prevent POI in patients undergoing colorectal surgery seems easily feasible as safety of ATB-346 was already shown in a phase 1 study in healthy subjects and a phase 2 clinical trial demonstrated pain relief of ATB-346 in patients with osteoarthritis; in both studies whole blood COX activity was profoundly suppressed (Wallace et al., 2018).

## Data Availability

The datasets generated for this study are available on request to the corresponding author.

## Ethics Statement

This study was carried out in accordance with the recommendations of the most recent national legislation (Belgian Royal Decree, 29/05/2013) and European Directive (Directive 2010/63/EU). The protocol was approved by the Ethical Committee for Animal Experiments from the Faculty of Medicine and Health Sciences at Ghent University (ECD 16-48).

## Author Contributions

RL designed the study. JD performed the experiments and data analysis. Intestinal histological evaluation was done by LP and JD under supervision of AV. RL and JD interpreted the findings and prepared and completed the manuscript. All authors approved the final version of the manuscript.

### Conflict of Interest Statement

The authors declare that the research was conducted in the absence of any commercial or financial relationships that could be construed as a potential conflict of interest.

## Footnotes

^1^Clinicaltrials.gov. *To Compare the Gastrointestinal Safety of a 14-Day Oral Dosing Regimen of ATB-346 to Sodium Naproxen in Healthy Subjects* (2018). Retrieved October 10, 2018, from https://ClinicalTrials.gov/show/NCT03291418

## Abbreviations

ADHP: 10-acetyl-3,7-dihydroxy-phenoxazine
ANOVA: One-way analysis of variance
COX: Cyclo-oxygenase
FD70: Fluorescein-labeled dextran (70 kDa)
GC: Geometric center
HETAB: Hexadecyl-trimethylammonium bromide
H_2_S: Hydrogen sulfide
IFN: Interferon
i.g.: Intragastrically
IL: Interleukin
IM: Intestinal manipulation
i.p.: Intraperitoneally
MCP-1: Monocyte chemoattractant protein-1
MPO: Myeloperoxidase
NaHS: Sodium hydrosulfide
NSAID: Non-steroidal anti-inflammatory drug
POI: Postoperative ileus.
